# Characterization and phylogenetic analysis of the complete mitochondrial genome of *Eristalia cerealis* (Diptera: Syrphidae)

**DOI:** 10.1080/23802359.2020.1720545

**Published:** 2020-02-03

**Authors:** Jingyan Yan, Shou Feng, Pengfei Song, Ying Li, Wangyan Li, Daoxin Liu, Xiuting Ju

**Affiliations:** a College of Agriculture and Animal Husbandry, Qinghai University, Xining, China;; b State Key Laboratory of Plateau Ecology and Agriculture, Qinghai University, Xining, China;; c Qinghai Provincial Key Laboratory of Animal Ecological Genomics, Xining, China

**Keywords:** *Eristalia cerealis*, Syrphidae, mitogenome, syrphid fly

## Abstract

The complete mitochondrial genome of *Eristalia cerealis* was sequenced and reported here. The circle genome of the syrphid fly is 15,348 bp in length. There are 38 sequence elements including 13 protein coding genes, 22 tRNA genes, 2 rRNA genes, and a control region. The order of all elements was the same with that of *E. tenax*. With 2 species from Muscidae and Drosophilidae as outgroups, phylogenetic relationships of 10 Syrphidae species based on mitogenomes were in complete agreement with their taxonomic relationships based on morphological characteristics. Our result will provide more fundamental data to the development of the molecular systematics of Syrphidae.

The grey-stripe syrphid fly, *Eristalia cerealis* (Fabricius, 1805), belongs to the Eristalini tribe in family Syrphidae, one of the most diverse families in Diptera, including about 6000 described species worldwide (Thompson 2013). Syrphidae adults have been regarded as important pollinators all along. According to the study by Ssymank et al. (2008), locally, up to 80% of flowering plants can be visited by syrphid flies. Larvae of a great number of hoverfly species are predacious on various agricultural pests, for example, soft-bodied Hemiptera (especially aphids), larvae of other Diptera, Acari and so on (Rojo et al. 2003), and can be used as important biocontrol agents (Wojciechowicz-Żytko and Wnuk 2012). Despite of the great ecological and economic value, classification of this huge family is still confined to traditional morphological taxonomy and reliable identification can only be achieved by limited experienced taxonomists (Mielczarek and Tofilski 2018). To facilitate the species diagnosis, molecular biology and DNA barcoding studies on Syrphidae should be payed enough attention to. So far, mitogenomes of only a dozen of hoverfly species have been reported (Li and Li 2019). Here, we sequenced the complete mitogenome of *E. cerealis*, to provide more fundamental data to the development of the DNA barcoding system.

In this study, *E. cerealis* adults were collected from Xining Botanical Garden, Qinghai, China (36°37′36″N, 101°44′58″E) in September 2019 and kept in the Insect Collection of the Entomology Lab, College of Agriculture and Animal Husbandry, Qinghai University, Xining, China (accession number: YJY-2019-SYY001). After genomic DNA extraction, genomic sequencing was performed on the Illumina HiSeq Platform (Illumina, San Diego, CA) with a read length of 150 bp. The software MITObim (Hahn et al. 2013) was employed to assemble the mitogenome. The assembled sequence was then annotated using the web server MITOS (Bernt et al. 2013).

A circularized DNA assembly 15,348 bp in length was harvested and deposited in the GenBank with accession number of MN912823. Like reported Syrphidae mitogenomes, 37 genes are recognized in this mitogenome: 13 protein-coding genes (PCGs), 22 transfer RNA (tRNA) genes and two ribosomal RNA (rRNA) genes, as well as a control region(D-loop). Of all 38 sequence elements, 4 PCGs, 8 tRNA genes and 2 rRNA genes are located on the light strand, while others are on the heavy strand. The order of all elements distributed on the sequence is consistent with that of *E. tenax* (Li et al. 2017).

Based on mitogenomes assembled here or downloaded from GenBank, phylogenetic relationships of 10 Syrphidae species with 2 species from Muscidae and Drosophilidae as outgroups were resolved by means of Neighbor-joining (Figure 1). After aligned using MAFFT (Katoh and Standley 2013), the Neighbor-joining tree was built using MEGA7 (Kumar et al. 2016) with bootstrap set to 1000. Phylogenetic relationships indicated by the phylogenetic tree were in complete agreement with their taxonomic relationships based on morphological characteristics, while species from genera *Eristalia* and *Eristalinus*, which belong to the tribe Eristalini and subfamily Milesiinae, respectively went into an independent clade and then formed a monophyletic clade.

**Figure 1.**
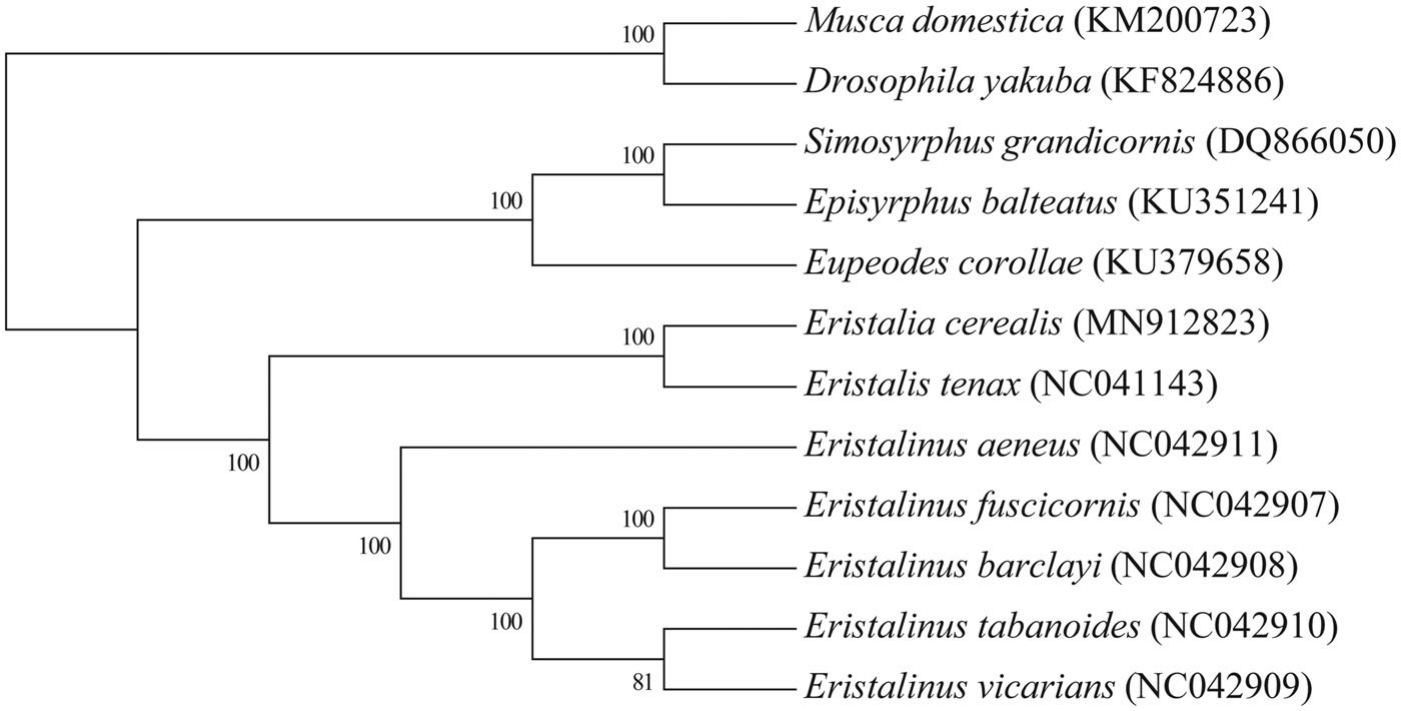
The Neighbor-joining tree based on 12 mitochondrial genome sequences.
